# Pseudo Differentiation Syndrome

**DOI:** 10.4084/MJHID.2011.061

**Published:** 2011-12-15

**Authors:** Dina Khalaf, Fathi Al-Jehani

**Affiliations:** 1 MBChB, MD, MSc, Hematology specialist; 2 MBChB, DIM, MSc, MRCP (UK), FRCPath, Consultant Hematologist

## Introduction

Differentiation syndrome (DS) is a life-threatening complication that may occur during the treatment of acute promyelocytic leukemia (APL) by all *trans*-retinoic acid (ATRA) therapy.^1^ Signs usually occur after 2 to 21 days of treatment and are generally an increase in the white blood cell count (WBC), fever, weight gain, dyspnoea, pleural effusion, and pulmonary infiltrates on chest radiograph. Some patients may develop renal failure, hypotension, and pericardial effusion.^2^

We are reporting what to our knowledge is the first case of DS in a relapsed refractory acute myeloid leukemia (AML) patient receiving decitabine.

## Case Report

44-year-old male patient with a relapsed refractory AML t(8;21). He had initially received multiple courses of chemotherapy. After his second complete remission he underwent a donor-matched allogenic peripheral stem cell transplant. The patient presented to our facility 16 months later with his second relapse. A bone marrow aspirate (BMA) and biopsy showed more than 50% myeloblasts Cytogenetics revealed Karyotype 46 XY, t(8;21)(q22;q22), t(4;20)(q22;q12), [19]/46,XY[1]. FLT-3 ITD was negative. Flowcytometry showed that blasts (CD45 dim) account for approximately 50% of the cells, CD13, CD33, CD34 and CD19 were positive. CD56 CD117 and HLADR were also positive. Treatment was initiated with decitabine (15 mg/m^2^ intravenous (IV) daily for 5 days repeated every 4 weeks) with palliative intent.

**Figure 1:**
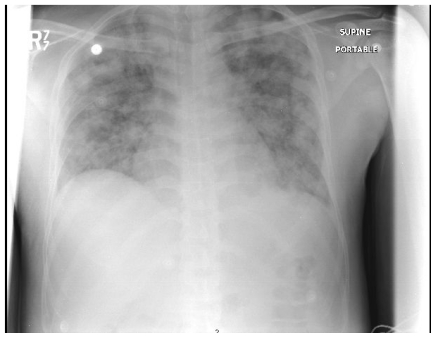
Chest X-ray showing bilateral lung infiltrates

Decitabine (5-aza-2′-deoxycytidine) is a hypomethylating agent which has demonstrated activity in Myelodysplastic Syndrome (MDS) and in secondary leukemia.^3^ The first two cycles were well tolerated. On the twelfth day of the third cycle, the patient presented with fever 39° C; chills; rigors; cough, dyspnea; and generalized edema. The patient looked ill, vital signs showed blood pressure 80/50, oxygen saturation 91% on room air. Lung auscultation revealed occasional fine basal crackles. There was bilateral lower limb pitting edema but no apparent septic foci. Complete blood counts (CBC) showed total white cell count (TLC) 5,400/uL; haemoglobin: 10/dL, platelet count 5,000/uL and neutrophils 3,200/uL. Blood, urine and sputum cultures were all negative and a normal chest X-ray (CXR). An empirical broad-spectrum antimicrobial was started but no growth factors were prescribed. The patient was transferred to the intensive care unit due to marked deterioration in his condition. Chest auscultation revealed bilateral wide spread crackles. A repeat CXR showed bilateral areas of infiltrates with consolidation (Figure 1). CBC showed an increase in TLC 11,800/uL; ANC 5,800/uL; Hemoglobin: 8.1 g/dL and Platelet count 20,000/uL. Repeated pan cultures with each spike of fever results were negative. A high resolution CT scan of the lungs revealed bilateral patches of alveolar infiltrates and areas of consolidation mainly in the upper lobes, air broncho-gram was seen inside. No cavitations or ring shadows suggestive of fungal infections were observed.

**Figure 2:**
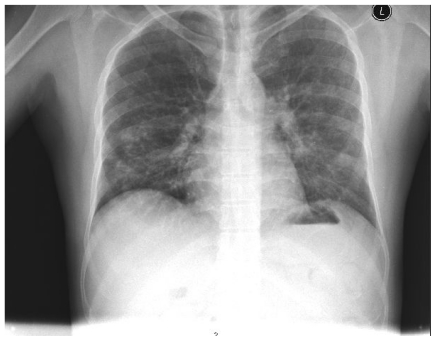
Chest X-ray showing marked improvement with steroid treatment

This clinical picture was highly suggestive of DS that is often seen in patients with APL on ATRA. Bronchoscopy and broncho-alveolar lavage (BAL) were recommended but the patient refused. Due to clinical concern that the patient was suffering a DS similar to the ATRA syndrome, Hydrocortisone 100 milligrams IV was given and lead to a dramatic improvement of his symptoms. He was maintained on dexamethasone (DXM) 10 mg IV twice daily with marked subjective, clinical and radiological improvement (Figure 2). The patient was discharged shortly afterwards when he was asymptomatic and clinically stable.

A pseudo-differentiation syndrome (PDS) was suspected in a patient with relapsing refractory AML, treated with decitabine with palliative intent. The patient’s clinical presentation, radiological findings; in addition to the absence of pre-existing lung pathology, negative repeated pan cultures as well as good response to DXM all favor DS. In the absence of biological diagnostic criteria of DS, diagnosis made on clinical grounds by the association of at least three of the following signs, in the absence of other causes: fever, weight gain, respiratory distress, lung infiltrates, pleural or pericardial effusion, hypotension, and renal failure.^1^ Our patient fulfilled five out of the eight mentioned criteria. Other conditions including bronchopneumonia in an immunocompromised patient, *Pneumocystis Jiroveci Pneumonia* (PCP) and fungal lung infection were ruled out. A trial of steroids was successful to control fever, dyspnea and constitutional symptoms, as well as reversing the CXR findings (**see** figures 1 and 2).

## Conclusions

This case supports the preliminary data that there might be biological activity of low dose decitabine in AML and suggests that clinical precautions similar to those implemented for the ATRA syndrome in ATRA-treated APL patients should be considered in Decitabine-treated AML with myeloid differentiation. The literature proved that patients with APL whose blasts express CD13 had higher risk to develop DS,^1^ this may initiate further research whether patients with AML with positive CD13 may carry higher risk to develop differentiation syndrome with hypomethylating agents.
